# Transporter-mediated L-glutamate elimination from cerebrospinal fluid: possible involvement of excitatory amino acid transporters expressed in ependymal cells and choroid plexus epithelial cells

**DOI:** 10.1186/s12987-015-0006-x

**Published:** 2015-04-29

**Authors:** Shin-ichi Akanuma, Tatsuhiko Sakurai, Masanori Tachikawa, Yoshiyuki Kubo, Ken-ichi Hosoya

**Affiliations:** Department of Pharmaceutics, Graduate School of Medicine and Pharmaceutical Sciences, University of Toyama, 2630 Sugitani, Toyama, 930-0194 Japan; Division of Membrane Transport and Drug Targeting, Graduate School of Pharmaceutical Sciences, Tohoku University, Aoba, Aramaki, Aoba-ku, Sendai, Miyagi 980-8578 Japan

**Keywords:** Excitatory amino acid transporter, EAAT1, EAAT3, Glutamine synthase, Ependymal cells, L-glutamate, Cerebrospinal fluid, Blood-cerebrospinal fluid barrier

## Abstract

**Background:**

L-Glutamate (L-Glu) is the major excitatory neurotransmitter in the CNS, and its level in cerebrospinal fluid (CSF) is reported to be increased in neuroexcitatory diseases such as epilepsy. Since L-Glu concentration in the CSF is reported to be lower than that in plasma, it has been proposed that some mechanisms of L-Glu clearance from the CSF operate in the brain. The purpose of this study was to elucidate the major pathway of L-Glu elimination from rat CSF and the transporters responsible.

**Methods:**

Protein expression and localization of excitatory amino acid transporters were examined by immunohistochemical analysis using specific antibodies. *In vivo* elimination of L-Glu from rat CSF was evaluated by intracerebroventricular administration. An L-Glu uptake study by using primary-cultured rat ependymal cells and isolated rat choroid plexus was performed to characterize L-Glu transport mechanisms.

**Results:**

An immunohistochemical analysis has shown that excitatory amino acid transporter (EAAT) 1 and EAAT3, which are D-aspartate-sensitive and kainate-insensitive L-Glu transporters, are localized on the CSF-side of rat ependymal cells and choroid plexus epithelial cells, respectively. In contrast, the kainate-sensitive L-Glu transporter, EAAT2, is not expressed in these cells. *In vivo* L-Glu elimination clearance from the rat CSF (189 μL/(min · rat)) was 23-fold higher than the CSF bulk flow rate, indicating that facilitative process(es) are involved in L-Glu elimination from the CSF. The *in vivo* [^3^H]L-Glu elimination from the CSF was significantly inhibited by unlabeled L-Glu and D-aspartate, but not kainate. Moreover, unlabeled L-Glu and D-aspartate inhibited [^3^H]L-Glu uptake by rat ependymal cells and choroid plexus epithelial cells, whereas kainate had little effect.

**Conclusion:**

It is suggested that EAAT1 in ependymal cells and EAAT3 in choroid plexus epithelial cells participate in L-Glu elimination from the CSF.

**Electronic supplementary material:**

The online version of this article (doi:10.1186/s12987-015-0006-x) contains supplementary material, which is available to authorized users.

## Background

L-Glutamate (L-Glu), an acidic amino acid, is regarded as the major excitatory synaptic neurotransmitter in the central nervous system (CNS) [1,2]. It has been known that L-Glu is mainly synthesized in neurons and released to the brain interstitial fluid [1,2], from where it may leak into the cerebrospinal fluid (CSF). In various CNS diseases, such as epilepsy, multiple sclerosis and Alzheimer’s disease, the L-Glu level in CSF, which fills the cerebral ventricles and bathes the brain, is reported to be increased [3,4]. Since excess accumulation of L-Glu leads to neural excitotoxicity [1], the L-Glu level in the CNS is considered to be strictly regulated. The L-Glu concentration in the rat CSF has been reported to be 11.4 μM, which is 14-fold lower than that in rat plasma [5]. Thus, it is conceivable that some mechanisms operate to eliminate L-Glu from the CSF and to regulate the brain/CSF L-Glu levels.

In general, it has been considered that there are three pathways for compound elimination from the CSF/cerebral ventricles [6,7]: (i) convective loss of compounds owing to CSF turnover, (ii) efflux transport across the blood-CSF barrier (BCSFB), which consists of choroid plexus epithelial cells (CPE cells), and (iii) uptake of the compound into the neural cells around the cerebral ventricles, such as ependymal cells. Convective loss, namely absorption of the compound into the circulating blood via the superior sagittal sinus, involves the passive and constant elimination of compounds with the CSF bulk flow rate (~2.9 μL/(min · rat)) [8]. In contrast, the other processes involve carrier-mediated and active elimination from the CSF. It has been reported that the transporters at the BCSFB contribute to the facilitative efflux transport of drugs and endogenous compounds from the CSF [9]. Regarding the carrier-mediated transport systems in ependymal cells, the expression and localization of some transporters, such as monocarboxylate transporters and urate transporter 1, has been studied, although a functional evaluation has not been fully achieved [10-12]. To consider the elimination of compounds including L-Glu from the CSF, it is important to evaluate the contribution of these processes to L-Glu clearance from the CSF and identify the molecule(s) responsible for this elimination.

It has been suggested that transporters which are expressed in CPE cells and ependymal cells contribute to L-Glu clearance from the CSF because some solute carrier (SLC) families are reported to be involved in the transport of L-Glu into neural cells. Excitatory amino acid transporters (EAATs), which are referred to as system X_AG_^−^, mediate the uptake of L-Glu and isomers of aspartate (Asp) [13]. To date, five distinct EAAT subtypes, EAAT1/GLAST/Slc1a3, EAAT2/GLT-1/Slc1a2, EAAT3/EAAC1/Slc1a1, EAAT4/Slc1a6, and EAAT5/Slc1a7, have been identified [14]. Also transporters selectively recognizing the L-isomer of Glu and Asp, alanine-serine-cysteine transporters 1 (ASCT1/Slc1a4) and ASCT2/Slc1a5, isoforms of system ASC, have been reported [14,15]. In addition, D/L-Asp-insensitive and Na^+^-independent system X_c_^−^, which is composed of xCT/Slc7a11 and 4F2hc/CD98/Slc3a2, is reported to be involved in the uptake of L-Glu and L-cystine [16-18]. The mRNA/protein expression of these transporters in ependymal cells and CPE cells has been reported [19-23]. However, the involvement of these transporters in the transport of L-Glu in CPE cells and ependymal cells from the CSF remains unknown. In particular, it has been found that the genetic deficiency of EAAT1-3 in rodents leads to neurologic dysfunction which is induced by excessive neuro-excitation and neurotoxicity [24-26]. Thus, there is a need for clarification of the role of EAAT1-3 in the homeostasis of L-Glu level in the brain, including L-Glu clearance from the brain/CSF.

The purpose of this study was to evaluate L-Glu elimination from rat CSF and clarify the transporters responsible in the cells around the cerebral ventricles. The protein expression and localization of EAAT1-3 in rat ependymal cells and CPE cells were investigated by immunohistochemical analysis. To elucidate the contribution of EAATs in these cells to the L-Glu transport process, we evaluated the elimination from the CSF following intracerebroventricular administration, and performed transport studies using isolated rat choroid plexus and primary-cultured rat ependymal cells.

## Methods

### Animals

Wistar rats (150-250 g) were purchased from Japan SLC (Hamamatsu, Japan). They were maintained in a controlled environment and all experiments were approved by the Animal Care Committee, University of Toyama.

### Reagents

L-Glutamic acid, [3,4-^3^H]- ([^3^H]L-Glu, 27.0 Ci/mmol) was obtained from Moravek Biochemicals (Brea, CA, USA). Mannitol, D-[1-^14^C] ([^14^C]D-mannitol, 55 mCi/mmol) and butanol, n [1-^14^C] ([^14^C]*n*-butanol, 2 mCi/mmol) were purchased from American Radiolabeled Chemicals (St. Louis, MO, USA). Rabbit anti-EAAT1 antibodies [27], which recognize EAAT1 and three kinds of splice variants (GLAST1a, GLAST1b, and GLAST1c [22]), and rabbit anti-EAAT2 antibodies [28], which recognize EAAT2 and do not bind to other splice variants (GLT-1b, exon 9-skipped GLT-1b, and GLT-1c [22]), were kindly gifted from Dr. M. Watanabe, Hokkaido University, Sapporo, Japan. All other chemicals were commercial products of analytical grade.

### Immunohistochemistry

Under deep pentobarbital anesthesia (100 mg/kg, i.p.), adult rats (n = 4) were killed by transcardial fixation with 4% paraformaldehyde in 0.1 M sodium phosphate buffer (PB, pH 7.4). The brains were isolated and immersed in 4% paraformaldehyde in 0.1 M PB and then 30% sucrose in 0.1 M PB. Frozen sections (20 μm thick) were prepared on a cryostat (CM1900; Leica, Nussloch, Germany). The sections were treated with 10% goat serum, and immunoreacted overnight with rabbit antibodies to EAAT1 (1.0 μg/mL; [27]), EAAT2 (1.0 μg/mL; [28]), EAAT3 (2.0 μg/mL; #EAAC11-A, Alpha Diagnostic International, San Antonio, TX, USA) and glutamine synthetase (GS, 1.0 μg/mL; [29]). Subsequently, they were incubated with rabbit-specific Cy3-conjugated secondary antibodies (Jackson ImmunoResearch, West Grove, PA, USA) at room temperature for 2 h. Nuclei were stained by incubating with 4 μM 4′,6-diamidino-2-phenylindole (DAPI) at room temperature for 5 min. The images were captured using a confocal laser scanning microscope (TCS-SP5; Leica). Fluorescence was detected with a confocal laser microscope equipped with a blue diode/green diode laser system. DAPI and Cy3 were excited sequentially using the 405 and 561 nm excitation laser wavelengths, respectively. Images were acquired using an appropriate pinhole to obtain 1 Airy unit. All images were acquired using confocal software (LAS AF, Leica), digitized at 8-bit resolution into an array of 1024 × 1024 pixels.

### *In vivo* L-Glu elimination from the CSF after intracerebroventricular administration

The elimination of compounds after intracerebroventricular administration was studied using the procedure described previously in detail [6]. Twenty-seven rats were anesthetized with an intraperitoneal injection of pentobarbital (50 mg/kg), and the head was fixed with a stereotaxic apparatus (SR-5R; Narishige, Tokyo, Japan). A hole was drilled in the skull, 1.5 mm left and 0.5 mm posterior to bregma, into which a needle was fixed as a cannula for injection. [^3^H]L-Glu (0.4 μCi, 15 pmol) and [^14^C]D-mannitol (0.01 μCi, 180 pmol) were dissolved in 10 μL extracellular cellular fluid (ECF) buffer (122 mM NaCl, 25 mM NaHCO_3_, 3 mM KCl, 1.4 mM CaCl_2_, 1.2 mM MgSO_4_, 0.4 mM K_2_HPO_4_, 10 mM D-glucose, and 10 mM HEPES, pH 7.4) and administered to the left lateral ventricle (0.5 mm posterior and 1.5 mm lateral to bregma; depth 4.0 mm). For inhibition studies, 50 mM unlabeled L-Glu, 25 mM D-Asp, or 12.5 mM kainate was administered simultaneously. Because it has been reported that the volume of rat CSF is 250 μL [30], the injected compounds after the intracerebroventricular administration (10 μL) were assumed to be diluted 25-fold. At designated times, CSF (50 μL) was withdrawn by cisternal puncture. Levels of ^3^H and ^14^C in the CSF and injectate were measured in a liquid scintillation counter (AccuFLEX LSC-7400; Hitachi-Aloka Medical, Tokyo, Japan).

Since it is reported that compounds administered into the lateral ventricles are eliminated from the CSF with one-compartmental kinetics according to Eq. 1, the kinetic parameters for [^3^H]L-Glu and [^14^C]D-mannitol were determined from Eq. 2 using the non-linear least-squares regression analysis program, MULTI [31]:

1 $$ {C}_{CSF}(t)\kern0.5em =\kern0.5em \frac{\mathrm{Dose}}{V_{\mathrm{d},\mathrm{C}\mathrm{S}\mathrm{F}}}\kern0.5em \times \kern0.5em  \exp \kern0.5em \left(-{k}_{\mathrm{el},\mathrm{C}\mathrm{S}\mathrm{F}}\kern0.5em \times \kern0.5em t\right) $$

2 $$ \frac{C_{CSF}(t)}{Dose}\kern0.5em \times \kern0.5em 100\kern0.5em =\kern0.5em \frac{100}{V_{\mathrm{d},\mathrm{C}\mathrm{S}\mathrm{F}}}\kern0.5em \times \kern0.5em  \exp \kern0.5em \left(-{k}_{\mathrm{el},\mathrm{C}\mathrm{S}\mathrm{F}}\times \kern0.5em t\right) $$

where *C*_CSF_(*t*), *V*_d,CSF_ and *k*_el_ are the CSF concentration at time *t*, the volume of distribution in the CSF and the elimination rate constant from the CSF, respectively, of either [^3^H]L-Glu or [^14^C]D-mannitol. “*C*_CSF_(t)/dose × 100” in Eq. (2) expresses the percentage of the residual concentration of compound in the CSF normalized by the amount of injectate (% of dose/mL CSF). The apparent elimination clearance from the CSF (*CL*_el,CSF_) was obtained by multiplying *k*_el_ by *V*_d_. In the inhibition study, the ratio of the percentage of residual concentration of [^3^H]L-Glu to that of [^14^C]D-mannitol was evaluated in the absence or presence of each inhibitor.

### Preparation of primary-cultured rat ependymal cells

Ependymal primary cultures were prepared from the brains of 22 newborn rats by dissociating whole brains as described previously [32,33]. The cells were resuspended in MEMc (minimum essential medium (MEM) supplemented with 0.5 g/L fatty acid-free bovine serum albumin (BSA), 5 mg/L insulin, 10 mg/L transferrin), seeded on fibronectin-coated culture vessels, and cultured at 37°C in humidified 5% CO_2_-air. Two days after the cultivation, the culture medium was changed to MEMc containing 500 U/L thrombin and renewed every third day. The subsequent cell studies were performed at least 14 days after the cultivation. To confirm prepared cells as ependymal cells, scanning electron microscopy was performed. Primary-cultured rat ependymal cells, which were plated onto fibronectin-coated 35 mm dish (Corning Incorporated Life Sciences, Tewksbury, MA, USA), were prefixed with 2% glutaraldehyde in divalent cation-free phosphate-buffered saline (PBS (-)) for 1 h, rinsed at least twice with PBS(-), postfixed with 1% osmium tetroxide in PBS(-) for 1 min at 4°C, and dehydrated in a series of ethanol. The samples were frozen in *t*-butanol at 4°C, and lyophilized. The specimens mounted on stubs were coated with platinum and were observed with a scanning electron microscope (S-4500; Hitachi, Tokyo, Japan).

### Immunocytochemical analysis

Primary rat ependymal cells were cultured in fibronectin-coated 8-well culture slides (Corning Incorporated Life Sciences). The cultured cells were washed with PBS(-) and then fixed by 4% formaldehyde in PB (pH 7.2) for 20 min at room temperature. After treatment with 0.1% Triton X-100 in PBS (-) for 10 min, the cells were blocked with 10% goat serum and then incubated overnight at 4°C with polyclonal rabbit anti-EAAT1 antibody (1 μg/mL; [27]) in 0.1% Triton X-100/PBS(-). Subsequently, the cells were incubated with Cy3-conjugated anti-rabbit IgG (1:200; Jackson ImmunoResearch). Nuclei were stained with 4 μM DAPI. The method for capturing images is the same as that described in the “Immunohistochemical analysis” subsection.

### [^3^H]L-Glu uptake by primary-cultured rat ependymal cells

Primary-cultured rat ependymal cells on fibronectin-coated 24-well plates (Corning Incorporated Life Sciences) were washed with ECF buffer, and incubated with 0.5 μCi/mL (19 nM) [^3^H]L-Glu at 37°C for 10 min in the absence or presence of inhibitors. After the assay, the cells were rinsed 3 times with ice-cold ECF buffer, lysed in 1 M NaOH, and neutralized with 1 M HCl. The radioactivity derived from [^3^H]L-Glu was measured in a liquid scintillation counter (AccuFLEX LSC-7400). A DC protein assay kit (Bio-Rad, Hercules, CA, USA) with BSA as a standard was employed to quantify the amount of cellular protein. The cellular uptake of [^3^H]L-Glu was evaluated as the cell/medium ratio according to Eq. 3.

3 $$ \mathrm{Cell}/\mathrm{medium}\ \mathrm{ratio}\ \left(\upmu \mathrm{L}/\mathrm{mg}\ \mathrm{protein}\right)\kern0.5em =\kern0.5em \frac{{}^3\mathrm{H}\hbox{-} \mathrm{Radioactivity}\ \mathrm{per}\ \mathrm{cell}\ \mathrm{protein}\ \left(\mathrm{dpm}/\mathrm{mg}\ \mathrm{protein}\right)}{{}^3\mathrm{H}\hbox{-} \mathrm{Radioactivity}\ \mathrm{concentration}\ \mathrm{in}\ \mathrm{medium}\ \left(\mathrm{dpm}/\upmu \mathrm{L}\right)} $$

### L-Glu uptake using freshly isolated rat choroid plexus

The uptake of [^3^H]L-Glu by rat choroid plexus was examined using the centrifugal filtration method described previously with minor modifications [34]. Thirty-one rats were decapitated, and then the choroid plexus was isolated from the lateral ventricles and pre-incubated at 37°C for 1 min in 100 μL ECF buffer. To initiate [^3^H]L-Glu uptake, pre-incubated choroid plexus was transferred to ECF buffer containing [^3^H]L-Glu (0.2 μCi/sample) and [^14^C]*n*-butanol (0.02 μCi/sample) in the absence (control) or presence of inhibitors. [^14^C]*n*-Butanol was used as a marker of the water space of the rat choroid plexus. The final concentrations of [^3^H]L-Glu and [^14^C]*n*-butanol were selected as 74 nM and 100 μM, respectively. The radioactivity of ^3^H and ^14^C was determined using a liquid scintillation counter (AccuFLEX LSC-7400). The tissue/medium concentration ratio was calculated from Eq. 4.

4 $$ \begin{array}{c}\hfill \mathrm{Tissue}/\mathrm{medium}\ \mathrm{ratio}\hfill \\ {}\hfill \left(\upmu \mathrm{L}/\upmu \mathrm{L}\ \mathrm{choroid}\ \mathrm{plexus}\right)\hfill \end{array}\kern0.5em =\kern0.5em \frac{\frac{\mathrm{Amount}\ \mathrm{of}\ \left[{}^3\mathrm{H}\right]\mathrm{L}\hbox{-} \mathrm{G}\mathrm{l}\mathrm{u}\ \mathrm{accumulated}\ \mathrm{in}\ \mathrm{the}\ \mathrm{choroid}\ \mathrm{plexus}\ \left(\mathrm{dpm}/\mathrm{the}\ \mathrm{choroid}\ \mathrm{plexus}\right)}{\left[{}^3\mathrm{H}\right]\mathrm{L}\hbox{-} \mathrm{G}\mathrm{l}\mathrm{u}\ \mathrm{concentration}\ \mathrm{in}\ \mathrm{the}\ \mathrm{medium}\ \left(\mathrm{dpm}/\upmu \mathrm{L}\right)}}{\frac{\mathrm{Amount}\ \mathrm{of}\ \left[{}^{14}\mathrm{C}\right]n\hbox{-} \mathrm{butanol}\ \mathrm{accumulated}\ \mathrm{in}\ \mathrm{the}\ \mathrm{choroid}\ \mathrm{plexus}\ \left(\mathrm{dpm}/\mathrm{the}\ \mathrm{choroid}\ \mathrm{plexus}\right)}{\left[{}^{14}\mathrm{C}\right]n\hbox{-} \mathrm{Butanol}\ \mathrm{concentration}\ \mathrm{in}\ \mathrm{the}\ \mathrm{medium}\ \left(\mathrm{dpm}/\upmu \mathrm{L}\right)}} $$

### Data analysis

The kinetic parameters, such as *V*_d,CSF_, *k*_el_, and *CL*_el,CSF_, determined by the least-squares regression analysis are presented as the mean ± SD. Other data represent the mean ± SEM. Statistical significance of differences between the means was determined by using the unpaired two-tailed Student’s *t*-test for two groups and one-way analysis of variance followed by Dunnett’s test for more than two groups. A value of *p* < 0.05 was accepted as statistically significant.

## Results

### Immunohistochemical analysis of EAAT1, EAAT2, EAAT3 and GS expression in the cells around the rat lateral ventricle

**Figure 1:**
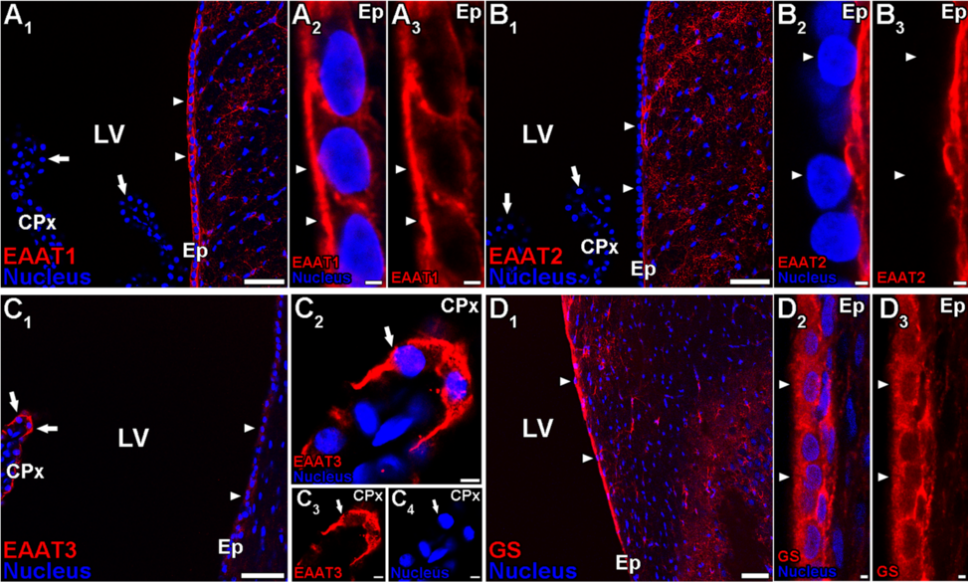
Immunohistochemical localization of EAAT1, EAAT2, EAAT3 and GS in neural cells around the rat lateral ventricle. Frozen sagittal sections from 6-week-old rats were stained with anti-EAAT1 **(A)**, anti-EAAT2 **(B)**, anti-EAAT3 **(C)**, or anti-GS **(D)** antibodies (red). The nucleus was visualized by incubating with DAPI (blue). **A**, EAAT1 immunoreactivity (red) was observed in the cells lining the lateral ventricle (LV) (arrow heads, **A**_**1**_), but not in the choroid plexus epithelial cells (CPx, arrows). At high magnification **(A**_**2**_-**A**_**3**_ **)**, EAAT1 was localized on the apical membrane (arrow heads) of the ependymal cells (Ep). **B**, EAAT2 immunoreactivity (red) was not observed in the cells lining the LV (arrow heads, **B**_**1**_) and choroid plexus epithelial cells (arrows, **B**_**1**_) although it was detected in the parenchymal cells in the brain. At high magnification **(B**_**2**_-**B**_**3**_ **)**, EAAT2 was not localized on the apical membrane (arrow heads) of the ependymal cells. **C**, EAAT3 immunoreactivity (red) was strongly detected in the choroid plexus epithelial cells (arrows) in the brain compared with the ependymal cells (arrow heads). At high magnification **(C**_**2**_-**C**_**4**_ **)**, EAAT3 was localized on the apical membrane (arrows) of the choroid plexus epithelial cells. **D**, GS immunoreactivity (red) was observed in the cells lining the LV (arrow heads, **D**_**1**_) as well as the parenchymal cells in the brain. At high magnification **(D**_**2**_-**D**_**3**_ **)**, GS was expressed in the ependymal cells (arrow heads). Scale bar: 50 μm **(A**_**1**_, **B**_**1**_, **C**_**1**_, **D**_**1**_ **)**, 2 μm **(A**_**2**_-**A**_**3**_, **B**_**2**_-**B**_**3**_, **D**_**2**_-**D**_**3**_), 5 μm **(C**_**2**_-**C**_**4**_ **)**.

The expression and localization of EAAT1-3 proteins in the cells around the lateral ventricle were examined by immunohistochemical analysis using specific antibodies for these transporters. Immunoreactivities derived from EAAT1, EAAT2, and EAAT3 were detected in neural cells of the brain parenchyma (red, Figure 1A-C). Immunostaining of EAAT1 (red, Figure 1A) was observed in the layer lining the lateral ventricles (arrow heads), whereas that of EAAT2 and EAAT3 (red, Figure 1B and C) was not detected in this layer. At higher magnification, the EAAT1 immunoreactivities were detected on the CSF side of the cells (arrow heads, Figure 1A2-A3), indicating that EAAT1 is localized on the apical membrane of ependymal cells. In contrast, the EAAT2 immunoreactivities were not detected on the CSF side of the cells (arrow heads) but on the brain side around the nucleus (Figure 1B2-B3). This result suggests that EAAT2 may be localized on the basal membrane of ependymal cells or on the end-feet of neural cells, such as astrocytes. L-Glu is metabolized to L-glutamine (L-Gln), an inactive substance, via glutamine synthetase (GS) [35]. The GS antibody resulted in the labeling of intracellular compartments of the ependymal cells (arrow heads, Figure 1D2-D3).

In rat CPE cells, EAAT3 immunoreactivities were very high (arrows) compared with ependymal cells (arrow heads, Figure 1C1). In contrast, immunostaining of EAAT1 and EAAT2 was not observed in CPE cells (arrows, Figure 1A1 and B1). At a high magnification, the EAAT3 immunoreactivities were detected on the CSF side/apical membrane of the CPE cells (Figure 1C2-C4, arrows).

### Elimination of [^3^H]L-Glu from rat CSF after intracerebroventricular administration

**Figure 2:**
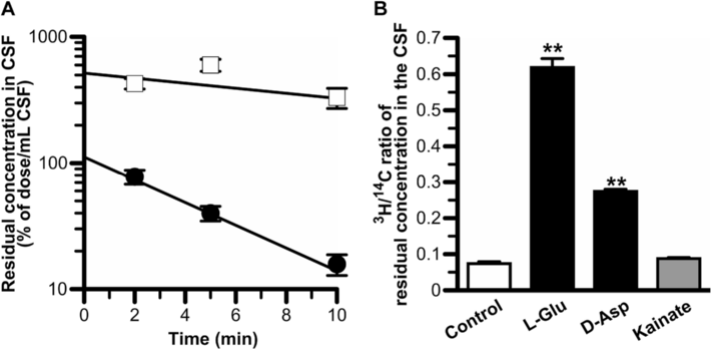
*In vivo* elimination of [^3^H]L-Glu from rat CSF. **A**. Residual concentration in rat CSF versus time profiles of [^3^H]L-Glu (closed circle) and [^14^C]D-mannitol (open square) after intracerebroventricular administration. The solution (10 μL) containing [^3^H]L-Glu (1.5 μM) and [^14^C]D-mannitol (18 μM) was administered into the rat lateral ventricle. The solid line was obtained using non-linear least-squares regression analysis. Each point represents the mean ± SEM (*n* = 3-4). **B**. Inhibition of [^3^H]L-Glu elimination from the CSF by simultaneous injection of unlabeled L-Glu (50 mM), D-Asp (25 mM), and kainite (12.5 mM). The concentrations in rat CSF of L-Glu, D-Asp, and kainate were estimated to be 2 mM, 1 mM, and 0.5 mM, respectively. The values, determined 5 min after intracerebroventricular administration, are expressed as the ratio of ^3^H/^14^C in the CSF divided by the same ratio in the injectate. Each column represents the mean ± SEM (*n* = 3-6). **p < 0.01, significantly different from the control.

Figure 2A shows the residual CSF concentration of [^3^H]L-Glu and [^14^C]D-mannitol after intracerebroventricular administration as a function of time. [^3^H]L-Glu was eliminated from the CSF with an elimination rate constant (*k*_el,CSF_) of 0.208 ± 0.032 min^−1^. This value was 4.5-fold greater than that of [^14^C]D-mannitol (0.0460 ± 0.0266 min^−1^), which is a reference compound for CSF turnover and diffusion into the brain interstitial space through the ependymal layer. The volumes of distribution in the CSF (*V*_d,CSF_) of [^3^H]L-Glu and [^14^C]D-mannitol were found to be 894 ± 131 μL/rat and 193 ± 131 μL/rat, respectively. The apparent elimination clearance (*CL*_el,CSF_) of [^3^H]L-Glu from the CSF (189 ± 34 μL/(min · rat)) was 20.9-fold greater than that of [^14^C]D-mannitol (8.89 ± 5.38 μL/(min · rat)). The elimination clearance of [^14^C]D-mannitol was in agreement with that in a previous report [6], and close to the CSF bulk flow rate (2.9 μL/(min · rat)) which was obtained by Suzuki *et al*. [8].

Following co-administration of unlabeled L-Glu (50 mM) into rat lateral ventricle, the ^3^H/^14^C ratio of the residual concentration in the CSF at 5 min was 8.4-fold greater than that in the control (Figure 2B). In addition, the simultaneous injection of 25 mM D-Asp with [^3^H]L-Glu resulted in a 3.7-fold increase in this ratio compared with that in the control, whereas co-administration of 12.5 mM kainate with [^3^H]L-Glu had little effect (Figure 2B). These results indicate that [^3^H]L-Glu elimination from rat CSF is inhibited in the co-presence of unlabeled L-Glu and D-Asp but not kainate.

### Expression of EAAT1 protein in primary-cultured rat ependymal cells

**Figure 3:**
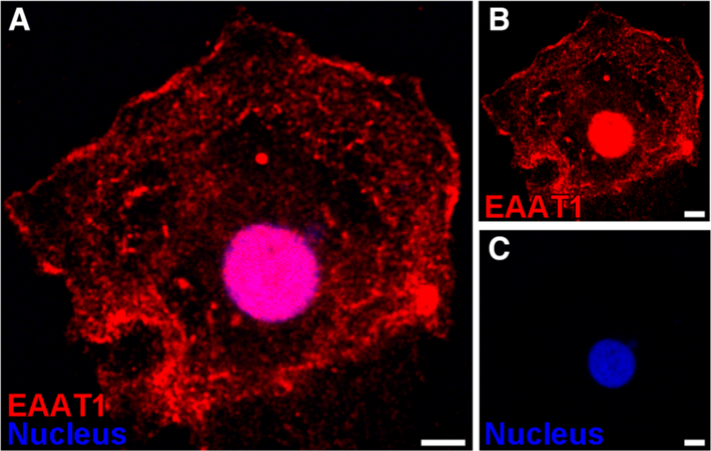
Immunostaining of EAAT1 in primary-cultured rat ependymal cells. Two-week-old primary-cultured rat ependymal cells were incubated with anti-EAAT1 antibodies (red, **A** and **B**). Nuclei were stained with DAPI **(A** and **C)**. Scale bar: 5 μm.

To investigate the existence of D-Asp-sensitive and kainate-insensitive L-Glu transport systems in ependymal cells, the protein expression of EAAT1, which is a D-Asp-sensitive and kainate-insensitive transporter, in primary-cultured rat ependymal cells, was examined. The cilia-like morphology was observed in 2-week-old ependymal culture by scanning electron microscopy. This is illustrated in (Additional file 1: Figure S1, Inspection of primary-cultured rat ependymal cells by scanning electron microscopy), indicating the validity of primary-cultured rat ependymal cells for this study. In immunocytochemical studies using anti-EAAT1 antibodies, immunostaining of EAAT1 was observed in primary-cultured cells (Figure 3). The EAAT1 immunoreactivities were found to be high not only in the nucleus (Figure 3A-C) but also in the plasma membrane of the cell (Figure 3A and B).

### L-Glu uptake by primary-cultured rat ependymal cells

**Figure 4:**
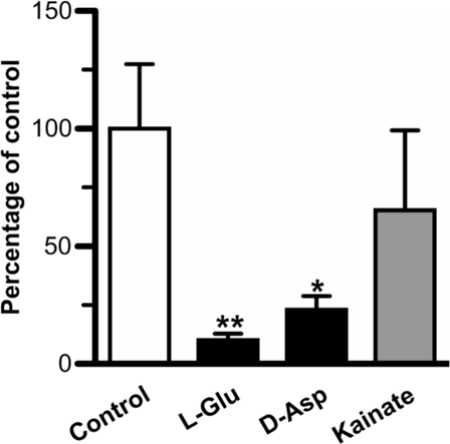
Effect of compounds on [^3^H]L-Glu uptake by primary-cultured rat ependymal cells. [^3^H]L-Glu (0.5 μCi/mL, 19 nM) uptake by 2-week-old primary-cultured rat ependymal cells was performed at 37°C for 10 min in the absence (control) or presence of unlabeled L-Glu (1 mM), L-Asp (1 mM), or kainate (0.5 mM). Each column represents the mean ± SEM (*n* = 3-6). *p < 0.05, **p < 0.01, significantly different from the control.

To examine the L-Glu transport mechanism(s) in ependymal cells, the effect of compounds on [^3^H]L-Glu uptake by primary-cultured rat ependymal cells was tested. [^3^H]L-Glu uptake by primary-cultured rat ependymal cells was significantly inhibited by 90% and 77% in the presence of unlabeled L-Glu and D-Asp, respectively (Figure 4). On the other hand, 0.5 mM kainate had little effect on [^3^H]L-Glu uptake by primary-cultured rat ependymal cells (Figure 4).

### Characteristics of L-Glu uptake by isolated rat choroid plexus

**Figure 5:**
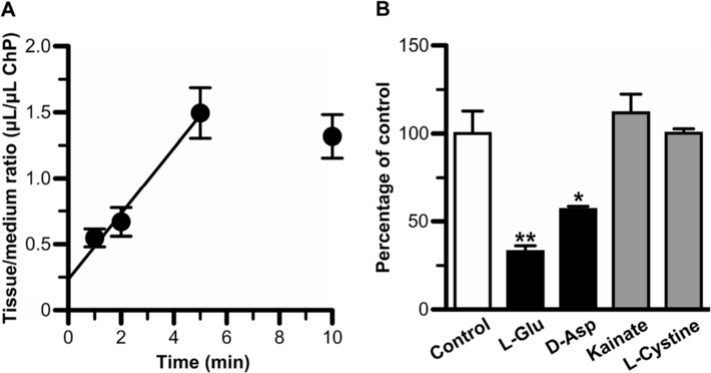
Characteristics of [^3^H]L-Glu transport at the blood-cerebrospinal fluid barrier. Isolated rat choroid plexus was incubated at 37°C with [^3^H]L-Glu (74 nM) and [^14^C]*n*-butanol (100 μM), which was used to determine the cell volume of the choroid plexus. **A**. Time-course of [^3^H]L-Glu uptake by isolated rat choroid plexus. Each point represents the mean ± SEM (*n* = 3-4). **B**. Effect of compounds on [^3^H]L-Glu uptake by isolated rat choroid plexus. Choroid plexus was incubated with [^3^H]L-Glu for 5 min in the absence (control) or presence of unlabeled L-Glu (1 mM), D-Asp (1 mM), kainate (0.5 mM), or L-cystine (0.25 mM). Each column represents the mean ± SEM (*n* = 3-4). *p < 0.05, **p < 0.01, significantly different from the control.

To examine whether L-Glu was eliminated from the CSF via the BCSFB, a [^3^H]L-Glu uptake study using isolated rat choroid plexus was performed. [^3^H]L-Glu uptake by isolated rat choroid plexus exhibited a time-dependent increase linearly for up to 5 min incubation with an initial uptake rate of 0.249 ± 0.047 μL/(min · μL choroid plexus) (Figure 5A). Since the volume of total choroid plexus has been reported to be 6 μL/rat [6], the elimination clearance of L-Glu from rat CSF across the BCSFB was estimated to be 1.49 ± 0.28 μL/(min · rat) by multiplying the initial uptake rate of [^3^H]L-Glu by the volume of total choroid plexus. Figure 5B shows the effect of compounds on [^3^H]L-Glu uptake by isolated rat choroid plexus. Unlabeled L-Glu (1 mM) significantly inhibited [^3^H]L-Glu uptake by isolated rat choroid plexus by 67%, indicating the involvement of carrier-mediated process(es) in L-Glu transport across the apical membrane of the BCSFB. [^3^H]L-Glu uptake by isolated rat choroid plexus was significantly inhibited by 43% in the presence of 1 mM D-Asp. In contrast, [^3^H]L-Glu uptake was not significantly altered in the presence of 0.5 mM kainate, which selectively inhibits EAAT2, or 0.25 mM L-cystine, which is a substrate of xCT.

## Discussion

In the present study, it was found that EAAT1 and EAAT3 were localized on the CSF side of ependymal cells and CPE cells, respectively (Figure 1). Based on these findings, the involvement of these L-Glu transporters in the elimination of L-Glu from the CSF was investigated by evaluating *in vivo* L-Glu elimination from the CSF (Figure 2) and transport studies using primary-cultured rat ependymal cells (Figure 4) and isolated rat choroid plexus (Figure 5).

[^3^H]L-Glu injected into the rat lateral ventricle was rapidly eliminated from the CSF (*CL*_CSF,eff_ = 189 μL/(min · rat)) compared with [^14^C]D-mannitol (*CL*_CSF,eff_ = 8.89 μL/(min · rat); Figure 2A), and this [^3^H]L-Glu elimination was inhibited by the simultaneous injection of unlabeled 50 mM L-Glu (Figure 2B). Because it has been reported that the volume of rat CSF is 250 μL [30], the injected compounds after the intracerebroventricular administration (10 μL) would have been diluted 25-fold. Based on this assumption, the concentration of unlabeled L-Glu after the simultaneous injection was estimated to be 2 mM, which is high enough to inhibit EAATs, ASCTs, and xCT [36-42]. These results suggest the involvement of a saturable process in the elimination of L-Glu from the CSF. D-Asp is reported to be a substrate of EAATs with *K*_m_ of less than 100 μM [43,44], and not a substrate or an inhibitor of ASCTs and xCT [15,17]. D-Asp (25 mM; estimated CSF concentration = 1 mM) co-administration inhibited [^3^H]L-Glu elimination from the CSF. This result indicates that EAATs are involved in the *in vivo* L-Glu elimination from the CSF. In contrast, co-administration of kainate (12.5 mM) did not significantly alter [^3^H]L-Glu elimination. Under this condition, the kainate concentration in the CSF was estimated to be 500 μM. It has been reported that kainate inhibits EAAT2 with a *K*_i_ of 59 μM, but not EAAT1 and EAAT3 at concentrations of 1 mM [43]. Taking these pieces of evidence into consideration, it is suggested that EAAT1 and EAAT3 play a role in the *in vivo* elimination of L-Glu from the CSF.

Our *in vitro* transport studies imply that EAATs are involved in the uptake of L-Glu into CPE and ependymal cells from the CSF via carrier-mediated mechanisms. EAAT1, EAAT3, and xCT have been reported to be expressed in ependymal cells although their localization on ependymal cells has not been fully evaluated [22,19]. From our immunohistochemical study using rat brain sections (Figure 1A-C), we found that EAAT1 was strongly expressed in ependymal cells relative to CPE cells, and localized on the apical membrane (i.e. CSF side) of the ependymal cells (Figure 1A). Anti-EAAT1 antibodies used in this study also recognize splice variants of EAAT1, such as GLAST1a, GLAST1b, and GLAST1c. Lee *et al*. have indicated that GLAST1a and GLAST1c are strongly expressed on the apical membrane of the choroid plexus epithelial cells [22]. Taking these points into consideration, we suggest that EAAT1 and/or GLAST1b are expressed in ependymal cells, and the protein expression level of these molecules on the apical membrane of ependymal cells is greater than that of EAAT1/GLAST and GLAST1a-1c in choroid plexus epithelial cells. In contrast, the protein expression of EAAT2 and EAAT3 in rat ependymal cells was lower than that in brain parenchymal cells and CPE cells, respectively (Figure 1B and C). It has been reported that GLT-1b and exon 9-skipped GLT-1b are localized on the apical membrane of choroid plexus epithelial cells [22], implying that these transporters could be involved in L-Glu uptake by choroid plexus epithelial cells. The uptake of [^3^H]L-Glu by primary-cultured rat ependymal cells, which express EAAT1 protein (Figure 3), was significantly inhibited by unlabeled L-Glu (Figure 4), suggesting the presence of a carrier-mediated transport system for L-Glu in ependymal cells. Moreover, this L-Glu uptake was significantly inhibited in the presence of D-Asp, a substrate of EAAT1-5 and not a substrate/inhibitor of ASCT1-2 and xCT [15,17]. On the other hand, the uptake was not significantly altered in the presence of kainate, which is an inhibitor of EAAT2/GLT-1 (Figure 4). These results suggest a minor contribution of EAAT2, ASCT1-2, and xCT to L-Glu uptake by ependymal cells. Although a selective inhibitory effect of EAAT1 and GLAST1b on L-Glu uptake by ependymal cells has not been elucidated, it is implied that EAAT1 is, at least in part, involved in the L-Glu uptake from the CSF into ependymal cells.

Regarding the L-Glu transport in rat CPE cells, we have already reported that L-Glu is taken up into a conditionally-immortalized rat CPE cell line [45], but the contribution to the L-Glu transport from the CSF to the CPE cells has not yet been determined. Our immunohistochemical studies revealed that EAAT3 was localized on the apical membrane of choroid plexus epithelial cells (Figure 1C), whereas EAAT1 and EAAT2 were not strongly expressed in CPE cells compared with ependymal cells and/or brain parenchymal cells (Figure 1A and B). It has been reported that two splice variants of EAAT1 (GLAST1a and GLAST1c) and two splice variants of EAAT2 (GLT-1b and exon 9-skipped GLT-1b) are localized on the apical membrane of choroid plexus epithelial cells [22]. Because anti-EAAT1 antibodies also recognize GLAST1a and GLAST1c, the protein expression level of these splice variants in choroid plexus epithelial cells could be low compared with that in ependymal cells. As for other L-Glu transporters, it has been reported that xCT proteins are localized on the apical membrane of rat CPE cells, and mRNAs of ASCT1 and ASCT2 are expressed in the rat isolated choroid plexus [20,21,44,46]. [^3^H]L-Glu uptake by isolated rat choroid plexus was inhibited by unlabeled L-Glu and D-Asp, but not kainate (a selective inhibitor of EAAT2) and L-cystine (a substrate of xCT, *K*_m_ = 48 μM) (Figure 5B) [16,43]. In addition, ASCTs and xCT are reported to be D-Asp-insensitive transporters [15,17]. These results indicate that GLT-1b, exon 9-skipped GLT-1b, ASCTs, and xCT play a minor role in L-Glu transport at the apical membrane of choroid plexus epithelial cells although ASCTs and xCT are expressed in choroid plexus epithelial cells. Taking these points into consideration, it is indicated that EAAT3 is involved in L-Glu uptake by isolated rat choroid plexus. To elucidate the contribution of EAAT3 to L-Glu transport in rat CPE cells, further studies on selective EAAT3 inhibition are needed. Nevertheless, our study suggests that EAAT3 participates in the transport of L-Glu from the CSF to CPE cells.

From the kinetic analyses of *in vivo* L-Glu elimination from CSF and the *in vitro* L-Glu uptake study by isolated rat choroid plexus, it does not seem that the convective loss due to turnover of the CSF and efflux transport across the BCSFB are major pathways for the clearance of L-Glu from the CSF. As the elimination clearance of D-mannitol from the CSF was found to be 8.89 μL/(min · rat) (Figure 2A), only 5% of the total L-Glu elimination from the CSF (189 μL/(min · rat)) would reflect the CSF bulk flow and diffusion into the brain interstitial space. In addition, the BCSFB-mediated elimination clearance of L-Glu from the CSF was estimated to be 1.49 μL/(min · rat) from the initial uptake rate of L-Glu by the isolated rat choroid plexus (Figure 5A). This value is 1% of the total L-Glu elimination from the CSF, and extremely low compared with the estimated efflux clearance of compounds, such as benzylpenicillin and PGE_2_, which shows facilitative elimination via the transporters at the BCSFB [6,47,48]. Thus, it appears that the CSF turnover and the BCSFB-mediated L-Glu efflux transport from the CSF contribute to only ~5.5% of L-Glu elimination from the CSF. Other routes for L-Glu elimination from the CSF, the remaining ~95%, have to be considered. Uptake of L-Glu into the neural cells which face the CSF and express some L-Glu transporters is thought to be most likely. It has been reported that the processes of astrocytes, which show L-Glu uptake via EAAT1 and EAAT2, are assembled next to the CSF [49,50]. In addition, our study reveals L-Glu uptake by ependymal cells via EAATs (Figure 4) and the localization of EAAT1 on the CSF side of the ependymal cells (Figure 1A). Harandi *et al*. have reported that [^3^H]L-Glu-derived signals were strongly detected on the layer of ependymal cells after intracerebroventricular administration of [^3^H]L-Glu [51]. Although the surface area of the ependymal cells has reported to be 2 cm^2^ [52], which is 37.5-fold lower than that of choroid plexus epithelial cells (75 cm^2^) [53], it is suggested that EAAT1-mediated L-Glu uptake into ependymal cells contributes, at least in part, to the major pathway (~95%) of L-Glu elimination from the CSF.

Our immunohistochemical study revealed the expression of GS in ependymal cells (Figure 1D). This result implies that ependymal cells are involved not only in the elimination of L-Glu from CSF but also in the supply of L-glutamine to the brain through that L-Glu uptake. It has been reported that EAAT1 deficiency is linked to epileptic syndrome [54]. In epileptic patients, the ratio of L-glutamine/L-Glu in the cerebral cortex and hippocampus is reported to be decreased [55]. Therefore, it is possible that transport of EAAT1-mediated L-Glu from the CSF and then GS-mediated conversion to L-glutamine play a role in preventing epileptic seizures, and this should be taken into consideration as therapeutic targets for the treatment of epilepsy.

## Conclusions

Ependymal cells play an important role in the elimination of L-Glu from the CSF. Moreover, EAAT1 on the apical membrane of ependymal cells takes part in the removal of L-Glu from the CSF, thereby maintaining a low L-Glu concentration in the CSF compared with that in plasma. Our findings provide a novel insight into the role of the ependymal cells in the homeostasis of the cerebral L-Glu level and may help us understand the pathogenesis of neuroexcitatory diseases.

## Additional file

Additional file 1:
**Inspection of primary-cultured rat ependymal cells by scanning electron microscopy.**

## Abbreviations

ASCT: Alanine-serine-cysteine transporters
Asp: Aspartate
BBB: Blood-brain barrier
BCSFB: Blood-cerebrospinal fluid barrier
BSA: Bovine serum albumin
*C*_CSF_(*t*): CSF concentration at time *t*
*CL*_el,CSF_: Apparent elimination clearance
CSF: Cerebrospinal fluid
CNS: Central nervous system
CPE cells: Choroid plexus epithelial cells
DAPI: 4′,6-diamidino-2-phenylindole
EAAT: Excitatory amino acid transporter
ECF: Extracellular fluid
Glu: Glutamate
GS: Glutamine synthetase
*k*_el,CSF_: Elimination rate constant from the CSF
MEM: Minimum essential medium
PB: Sodium phosphate buffer
PBS (-): Divalent cation-free phosphate-buffered saline
SLC: Solute carrier
*V*_d,CSF_: Volume of distribution in the CSF
