# Supplementary material for: Transporter-mediated L-glutamate elimination from cerebrospinal fluid: possible involvement of excitatory amino acid transporters expressed in ependymal cells and choroid plexus epithelial cells
Source: Fluids Barriers CNS. 2015 Apr 29;12:11. doi: 10.1186/s12987-015-0006-x (PMC4425921; doi:10.1186/s12987-015-0006-x)
Supplement: Additional file 1: — Inspection of primary-cultured rat ependymal cells by scanning electron microscopy. [file 12987_2015_6_MOESM1_ESM.pdf]

Additional file 1

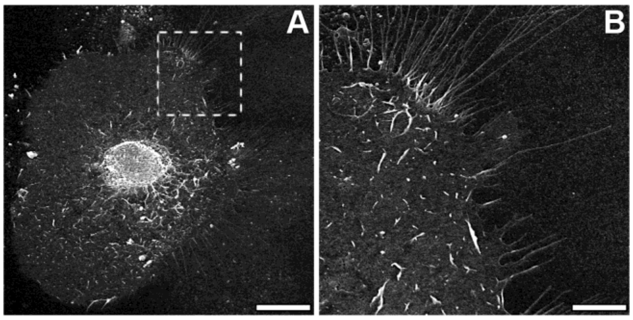

Figure S1 Inspection of primary-cultured rat ependymal cells by scanning electron microscopy. A, Micrographs of 2-week-old ependymal culture. B, Higher magnification of the boxed area in Supplemental Figure 1A. The cultured cells had cilia. Scale bar: 15 μm (A), 5 μm (B).
